# Supplementary material for: A set of multi-entry identification keys to African frugivorous flies (Diptera, Tephritidae)
Source: Zookeys. 2014 Jul 24;(428):97–108. doi: 10.3897/zookeys.428.7366 (PMC4143993; doi:10.3897/zookeys.428.7366)
Supplement: Supplementary material 4 — Key to Capparimyia [file zookeys-428-097-s004.zip › SF4_ZooKeys_key to Capparimyia/lucid_player/help/help/whgdata/whlstf0.htm]

Search Words List


**128mb**   
**166mhz**   
**32mb**   
**51mb**   
**70mb**   
**75mb**   
**85mb**   
  
  
**abdomen**   
**able**   
**above**   
**absent**   
access **1** **2** **3** **4** **5**   
**accessed**   
**accuracy**   
**accurate**   
activate **1** **2** **3**   
**activated**   
**activation**   
**actually**   
**add**   
addition **1** **2**   
address **1** **2** **3** **4** **5**   
addressed **1** **2**   
**addressing**   
**administered**   
**administrators**   
**again**   
**aims**   
**algorithm**   
allow **1** **2** **3** **4** **5** **6**   
allowing **1** **2**   
allows **1** **2** **3** **4**   
**almost**   
**along**   
**alongside**   
already **1** **2**   
**alternate**   
alternative **1** **2** **3**   
alternatively **1** **2**   
**although**   
**amongst**   
animals **1** **2**   
**annotated**   
another **1** **2**   
answer **1** **2** **3**   
**answered**   
**answers**   
**antennae**   
any **1** **2** **3** **4** **5** **6** **7** **8** **9** **10**   
**apparently**   
**appear**   
**apple**   
applet **1** **2** **3** **4**   
application **1** **2** **3** **4**   
**applied**   
applies **1** **2** **3**   
**applying**   
appropriate **1** **2** **3**   
**archaeological**   
arranged **1** **2** **3**   
**artefacts**   
**asian**   
**asked**   
associated **1** **2** **3**   
**assumes**   
**attach**   
attached **1** **2**   
**attachments**   
**attempting**   
author **1** **2** **3**   
**authors**   
auto **1** **2**   
**autobest**   
automate **1** **2** **3** **4**   
automated **1** **2** **3**   
automatically **1** **2**   
**automating**   
**autoprune**   
**availability**   
available **1** **2** **3** **4** **5** **6** **7** **8** **9** **10** **11** **12** **13**   
**average**   
  
  
back **1** **2**   
**background**   
bar **1** **2** **3** **4**   
based **1** **2**   
**basic**   
**basis**   
**beetles**   
before **1** **2**   
**behaviour**   
being **1** **2** **3** **4**   
**believe**   
**belong**   
below **1** **2** **3**   
best **1** **2** **3** **4** **5** **6** **7** **8**   
better **1** **2**   
**beyond**   
**bingo**   
blue **1** **2**   
**body**   
**books**   
both **1** **2** **3** **4** **5**   
bottom **1** **2**   
box **1** **2** **3** **4** **5** **6**   
boxes **1** **2** **3**   
**branches**   
**branching**   
**brazil**   
**browse**   
browser **1** **2** **3**   
build **1** **2** **3**   
builder **1** **2** **3** **4** **5** **6**   
**builder1**   
**built**   
**bundled**   
**butterflies**   
button **1** **2** **3** **4** **5** **6** **7** **8** **9** **10** **11** **12** **13** **14**   
buttons **1** **2** **3**   
  
  
**c**   
called **1** **2**   
cannot **1** **2** **3**   
**capturing**   
**careful**   
case **1** **2** **3** **4** **5** **6** **7** **8**   
cause **1** **2**   
**causes**   
cd **1** **2**   
**centrally**   
certain **1** **2**   
change **1** **2**   
**changes**   
changing **1** **2**   
**character**   
**characteristics**   
**characters**   
**charge**   
check **1** **2** **3** **4** **5**   
checkbox **1** **2** **3**   
**checkboxes**   
checked **1** **2** **3** **4** **5** **6**   
**choice**   
choose **1** **2** **3** **4** **5** **6** **7** **8** **9** **10** **11** **12**   
choosing **1** **2** **3** **4** **5** **6** **7** **8** **9** **10**   
**chose**   
chosen **1** **2** **3** **4** **5** **6** **7** **8** **9**

**>>**
